# Bibliometric review of visual computing in the construction industry

**DOI:** 10.1186/s42492-020-00050-0

**Published:** 2020-06-08

**Authors:** Heng-Wei Wang, Zhen-Zhong Hu, Jia-Rui Lin

**Affiliations:** 1 Tsinghua Holdings Company Limited, Beijing, 100084 China; 2 Department of Civil Engineering, Tsinghua University, Beijing, 100084 China; 3 Tsinghua Shenzhen International Graduate School, Tsinghua University, Shenzhen, 518055 China

**Keywords:** Visual computing, Bibliometric analysis, Construction application, Building information modeling, Laser scanning, Augmented reality, Construction management

## Abstract

In the construction area, visuals such as drawings, photos, videos, and 3D models, play a significant role in the design, build and maintenance of a facility, bringing efficiency to generate, transfer, and store information. Advanced visual computing techniques facilitate the understanding of design contents, work plans, and other types of information shared in the construction industry. Automatic visual data collection and analysis provide many possibilities to the construction industry and a large number of works have investigated how visual computing can improve construction management processes and other problems in the construction area. However, a comprehensive literature review is needed. This study uses bibliometric approaches to review the works published to date, and analyses the development of knowledge, significant research results, and trends. The purpose of this study is to help newcomers to this research field understand knowledge structure and formulate research directions, thereby enhancing knowledge development. From this study, it can be concluded that computer vision is a key axis of improvement. Moreover, building information modeling, laser scanning, and other visualization-related techniques are also important in advancing the construction area.

## Introduction

Visual computing is a general term that covers a broad research area for collecting and processing images, videos, three-dimension (3D) models, and other types of visual data [1]. It is significant to store, transfer, present, generate, and analyze visual information because people rely most on visual information to understand the outside world. Visual computing provides many possibilities for improving industries, such as the construction industry [2]. In the construction industry, engineers traditionally use drawings and documents to represent design information and use photos or texts to record on-site information. Nowadays, building information modeling (BIM) makes it possible to build an integrated database during the life cycle of construction projects [3]. In the construction phase, the 3D design model with related engineering information is used to explain, assist, and verify on-site works [4]. BIM provides methods to represent construction information via shared 3D scenes, which can reduce misunderstanding and promote communication between project stakeholders. BIM is closely associated with several visual computing techniques, including modeling and visualization of 3D models, real-time 3D animations, mobile 3D interaction, as-built modeling, and 3D- two-dimension (2D) synchronization. BIM has enabled the development of new construction approaches with high efficiency, and has provided many opportunities for applying computer technology to avoid redundant tasks in the traditional construction processes [5]. Meanwhile, vision-based processing and data mining are used to monitor construction sites and recognize key events automatically. While traditional approaches for monitoring construction site require manpower, cameras can record the condition of construction sites more continuously. In addition to fixed cameras, drones and other movable devices provide more comprehensive viewpoints. Meanwhile, real-time video processing and analysis approaches contribute to long-time monitoring and timely warning.

Although visual computing plays an essential role in construction, no bibliometric review has been provided untill now. Bibliometric methods are exhaustive and useful in analyzing all academic works in a given area [6]. Tools supporting bibliometric analysis and visualization based on reliable scientific literature sources, such as the Web of Science, Scopus, and Google Scholar, have been proposed and used for scientific review [7]. CiteSpace [8], VOSviewer [9], and SCI2 [10] are tools typically used for graph visualization, highlighting not only the potential patterns but also the scientific changes.

In order to discover the development path of the knowledge body of visual computing in construction, this study uses bibliometric tools to analyze the relevant literature with citation information. Based on the results, a further explanation of the scientific changes is proposed to highlight the contributors, their influences, main topics, and trends. This study aims to comprehensively dissect the research work on visual computing in construction, to help locating the critical knowledge and to indicate the recent trends and potentials to guide future investigations in this area.

## Methodology

**Fig. 1:**
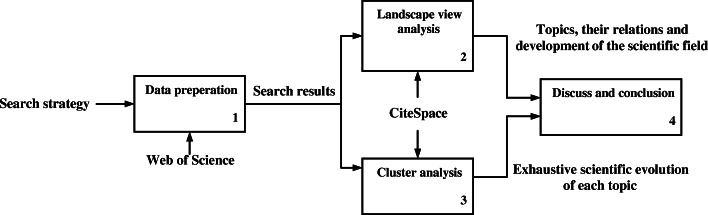
IDEF0 diagram for the process of this study

This study conducts a three-step bibliometric analysis of the research works in the area of visual computing in construction, as shown in Fig. 1. The first step is data preparation. In this step, a search strategy with different keywords is applied to obtain literatures and their details for bibliometric analysis. Based on the data collected in the first step, the research history and trends of the field are analyzed. CiteSpace is an evolving software for a systematic review of research fields [7] and it is one of the few bibliometric tools that support timeline analysis, so we choose it to generate the landscape co-citation network and the timeline view for trends analysis. By using CiteSpace, we generate and analyze the landscape view and clusters in the second and third steps. After the three-step bibliometric analysis, this study discusses the results and draws conclusions that will contribute to the body of knowledge.

## Bibliometric analysis

### Data preparing

**Fig. 2:**
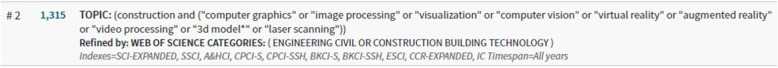
Search strategy used for searching database of Web of Science

Visual computing is a broad scientific area that includes multiple disciplines and research topics, such as computer graphics, image and video processing, 3D visualization, and virtual and augmented reality (AR) [11]. Laser scanning and 3D modeling are also key topics, especially in construction. This study chooses the Web of Science as the literature source to ensure the quality of the collected literatures. The search strategy is shown in Fig. 2.

The word visual computing is so broad and it is not directly used in most of the published papers. Therefore, several key research topics in visual computing are used in the search strategy of this study. This search was conducted on November 24, 2019. With the timespan not specified, it captures 1315 results. And all the results are used in the analysis and the conclusions drawn from it.

### Landscape views

**Fig. 3:**
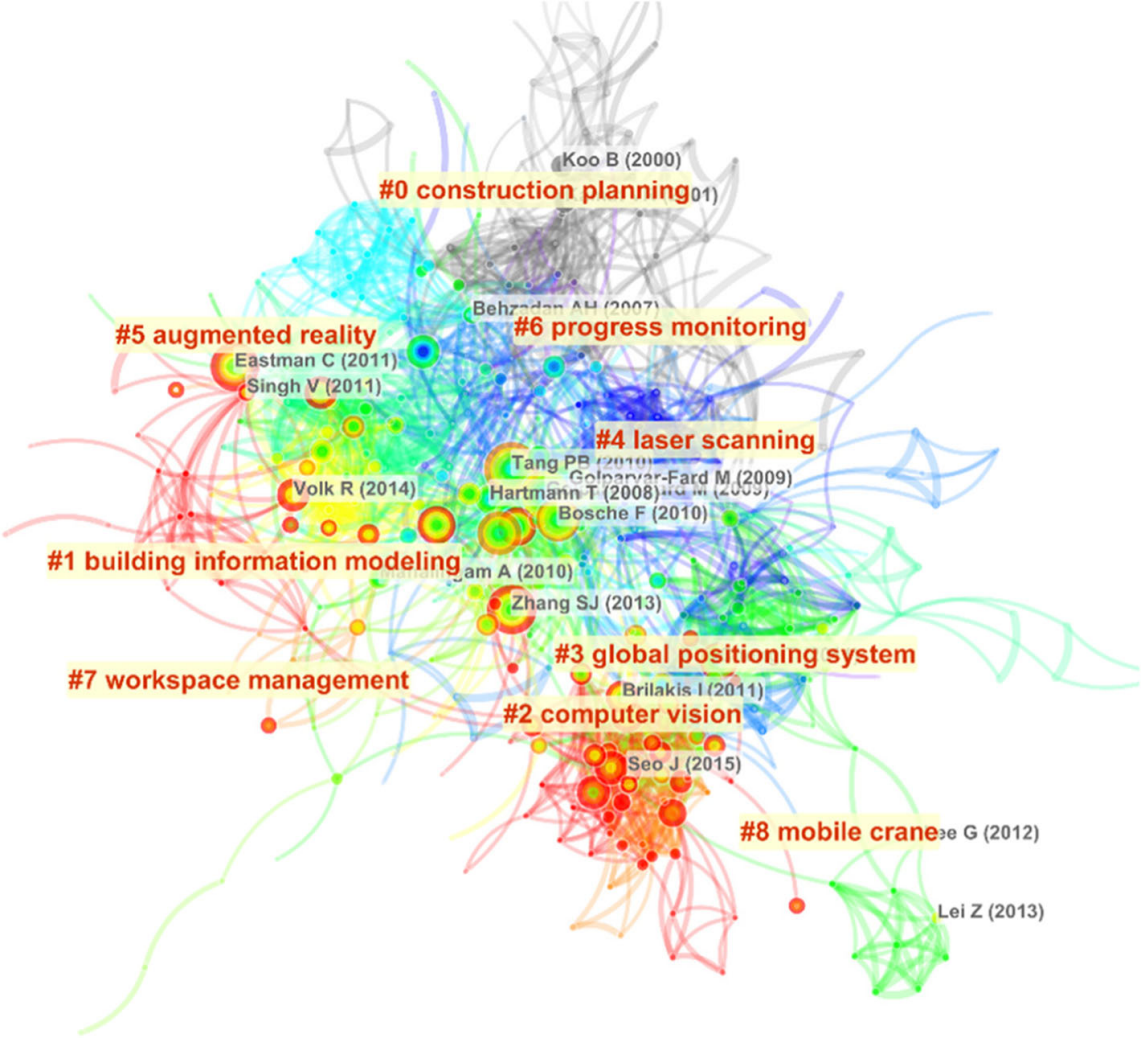
A landscape view of the co-citation network

A landscape view of the co-citation network obtained from the search results, for the period 1996–2019, is shown in Fig. 3. A clustered co-citation network shows the influence of each published work, their citation relationships, and their correlations. In the graph, the node colors show the influence of the published works. The warm colors (e.g., red) indicate a recent influence, and the cold colors (e.g., blue and gray) indicate an older influence. In Fig. 3, nine clusters are detected and labeled with keywords. Among them, cluster #4 and #6 are at the central position, indicating that these studies have a strong connection with others. Moreover, the node colors have a relatively concentrated distribution, indicating the fast evolution of knowledge.

**Fig. 4:**
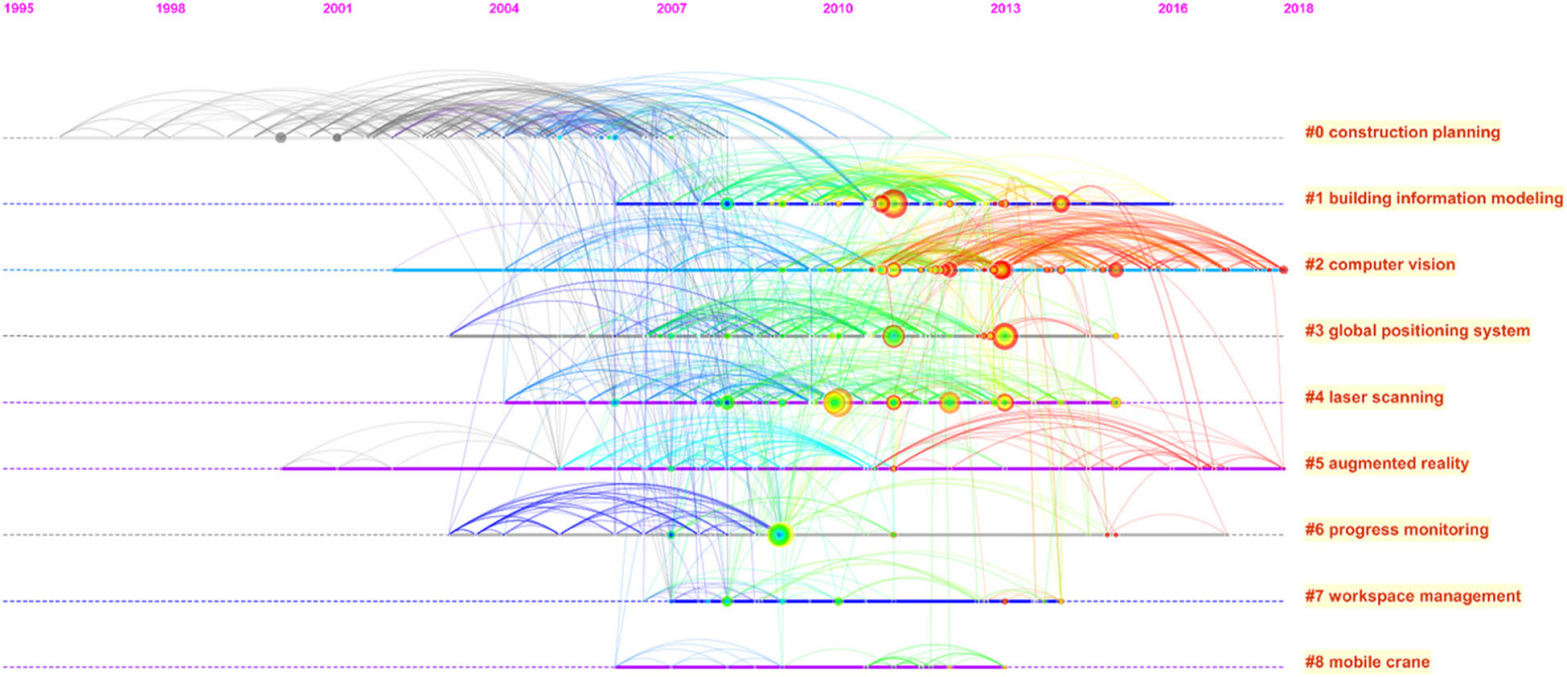
The landscape timeline view of the co-citation network

To briefly show the development history of each cluster, we generate a timeline view shown in Fig. 4. In the timeline view, the position of the nodes in the timeline refers to the year when the literature was published. Although our search scope contains the year 2019, the works published in 2019 are not contained in Fig. 4, because they have not been cited yet. Three types of clusters could be identified. The first type includes cluster #0. The main feature of this type is that the publishing years of nodes are relatively old, and the colors of nodes are mainly cold, which indicates that the publications associated with this type of cluster have almost no effect in recent years. The second type of cluster includes cluster #1, #2, #3, and #4. The main feature of this type is that a large number of nodes have a significant impact, and most of them are still important in recent years. The third type includes cluster #5, #6, #7, and #8. In this type of cluster, works have been published recently and it seems they would have a continuous influence on the research directions.

**Fig. 5:**
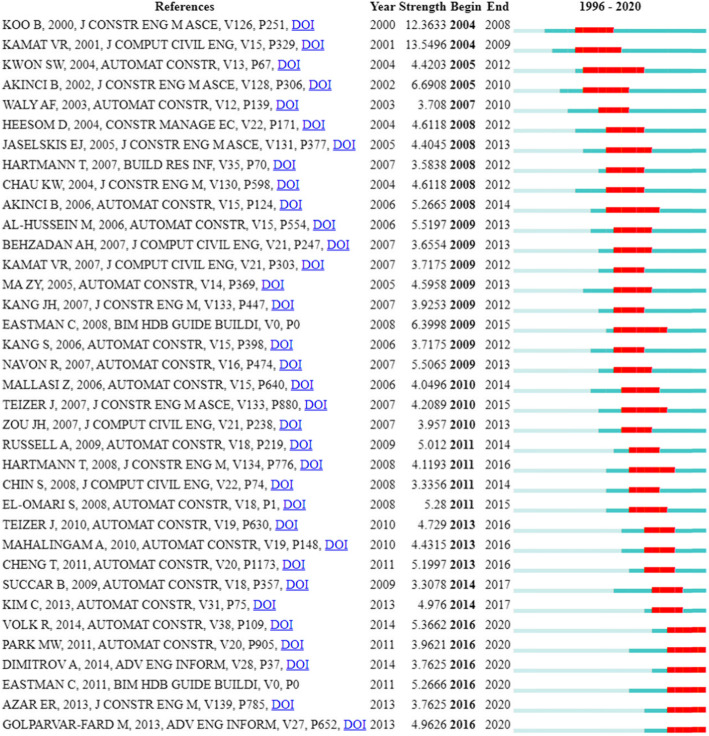
Top 36 burst references with citation bursts in at least 4 years

The research interests are changing continuously and some works have a significant influence on the changing of directions. We list the top 36 works with citation bursts of at least 4 years in Fig. 5. The reference with the largest strength was proposed by Kamat and Martinez [12]. This paper emphasizes the importance of 3D visualization of the construction operation simulation and implements the first prototype system. The most recent burst references include the works of BIM [13, 14] and the works of computer vision-based recognition and tracking [15–18]. This result demonstrates that BIM and computer vision are gaining burst attention in these years, which is consistent with the results presented in Fig. 4. Specifically, one of the mentioned BIM-related works is the second edition of the BIM handbook, and the other one is a literature review of BIM. They summarized the application and research directions of BIM, and provide a guideline for new researchers. While the burst works of computer vision mostly concentrate on some specific techniques, i.e., object recognition. The different results of the two above-mentioned research topics indicate that they have different burst reasons. In the section of research topic analysis, these reasons are revealed by a detailed analysis of the cluster data.

### Research topic analysis

**Fig. 6:**
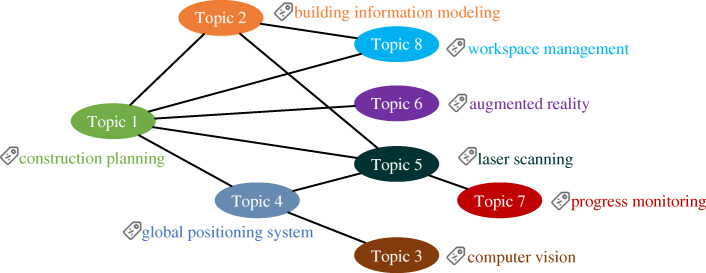
Significant citation relationships between topics

In the topic analysis, we start with topic number 1, which corresponds to cluster #0. Each topic has a label suggested by CiteSpace based on the log-likelihood ratio. In some cases, the suggested labels cannot represent the key topic properly. Therefore, before a detailed analysis, we use only the topic number. Despite the research topics being clustered, some of them have apparent citation relationships. When considering a number of citations over ten as a criterion to define significant relationship, we can find ten significant relationships, as shown in Fig. 6. The label of topic 1 is construction planning. The core technique of topic 1 is 3D computer-aided design (CAD). The relationship between topic 1 and topic 2 illustrates that BIM is used to support construction planning as 3D CAD does. Topic 1 and topic 2 are both related to topic 5 and topic 8 because workspace management and laser scanning are both related to construction planning, 3D modeling, and building information modeling. A significant difference between topic 5 and topic 8 is that laser scanning supports as-built 3D modeling, but workspace management uses scheduling data and building information modeling. Topic 1 also has significant citation relationships with topic 6 and topic 4, showing that AR and positioning techniques can support construction planning. Construction safety is the main problem mentioned in topic 4. Topic 4 is related to topic 5 and topic 3, reflecting that laser scanning and computer vision support construction safety. Topic 7 only has a significant relationship with topic 5, indicating that laser scanning is usually used for progress monitoring recently.

Meanwhile, Fig. 4 shows that works related to topic 1 have the highest citation number among all topics. Topics 3, 4, and 9 are the most closed topics in the searching view of this study.

#### Topic 1: construction planning

**Fig. 7:**
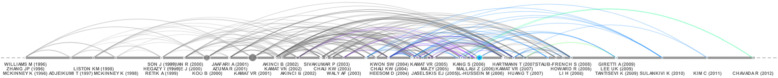
Timeline view of topic 1

**Fig. 8:**
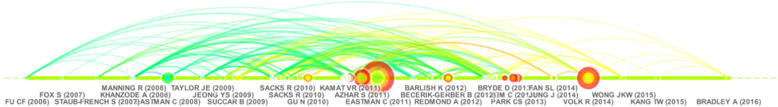
Timeline view of topic 2

The label of topic 1 is construction planning. It indicates the research direction in most cases. In the cluster of this topic, the top two cited works include the feasibility research of four-dimension (4D) CAD in commercial construction [19] and an approach enabling animated construction operations in 3D [12]. These two works illustrate that this topic concentrates on showing the construction progress in an animated 3D scene. Other typical works include integrating 3D visualization and simulation for tower crane operations [20], visualization of crane erection processes [21], 4D application for site layout and management [22], workspace analysis utilizing 4D visualization [23] and construction processes simulation systems [24]. The common purpose of all these works is to help people understand the construction process by simulating it in an intuitive scene. Figure 7 shows the timeline view of topic 1 with representative contributors. After 2010, it is hard to find more works in this cluster. The main reason could be that BIM replaced 3D CAD. BIM supports not only 3D modeling, like 3D CAD does, but also integrating project information in the entire project life-cycle. The timeline view of topic 2 (Fig. 8) shows that BIM became a research topic in this scientific field in 2007. After that, 3D CAD was gradually less mentioned.

**Fig. 9:**
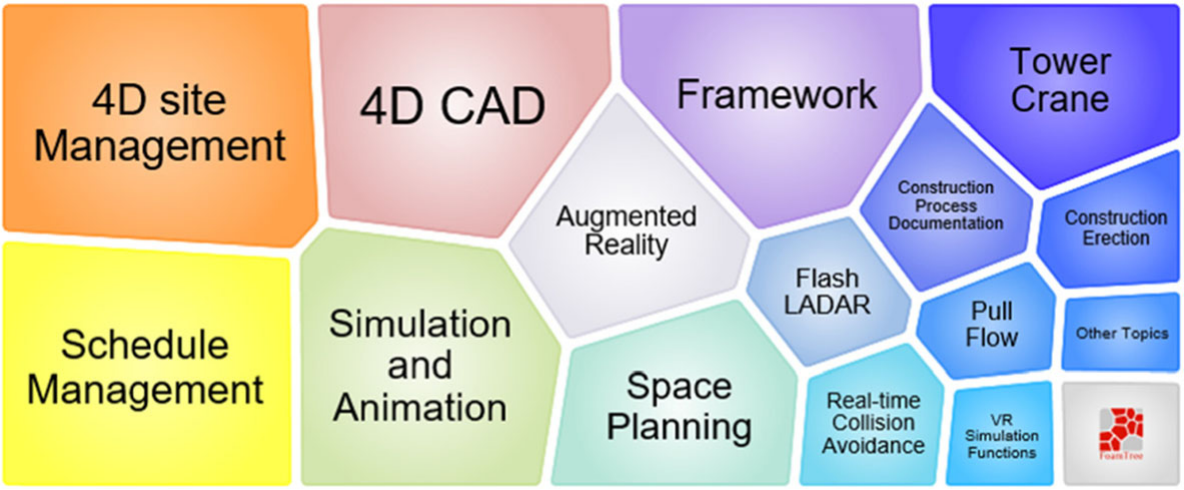
Foam tree view of keywords in topic 1

Figure 9 illustrates the foam tree of keywords in topic 1. The figure indicates that it focuses on 4D site management and schedule management. The techniques used in the cluster include 4D CAD, AR, and virtual reality (VR). They are used for simulation and animation to engage in construction control.

#### Topic 2: BIM

Topic 2 is labeled as BIM. When concentrating on the most cited works, we can pick out the core concept of this topic. The most cited work is the second edition of the BIM handbook [14]. It was published in 2011 and has 51 citations in all the collected literatures. The second cited work is the literature review of BIM [13]. The two above-mentioned works are also the most recent bursts found in Section 3.2, indicating that these two works are both the most important and the most popular in recent years in the topic. While both works focus on BIM, the BIM handbook introduces and explains how to use BIM, while the other work reviews the literature and suggests the future works on using BIM for existing buildings. Both works systematically describe BIM, in the same way as as the third and fourth cited works: an introduction of BIM [25] and the first edition of the BIM handbook [26].

Figure 8 shows that topic 2 started in 2006 when Fu et al. [27] proposed an industry foundation classes model viewer to support the nD model application [28]. Afterward, related works in this topic can be classified according to the research purpose. Some works aim to solve the BIM application problems, including the information requirement research [29], analyzing how to adopt BIM in the architecture, engineering, and construction (AEC) industry [30], and using BIM to support sustainable design [31] and construction [32]. Some other works aim to explain the drivers for using BIM, such as exploring how to measure the benefits of BIM [33] and analyzing the project benefits of BIM [3]. Meanwhile, some works aim to solve technical problems when using BIM, such as enhancing the information exchange process [34], information integration [35, 36], spatial data analysis [37, 38], and geometric optimization [39]. Furthermore, some works aim to enhance construction using BIM and other techniques, such as assisting the construction defect management [40], monitoring building performance [41], improving the monitoring of construction supply chain [42], using BIM and 4D to solve structural conflict problems [43, 44], and lean production management systems based on BIM [45].

**Fig. 10:**
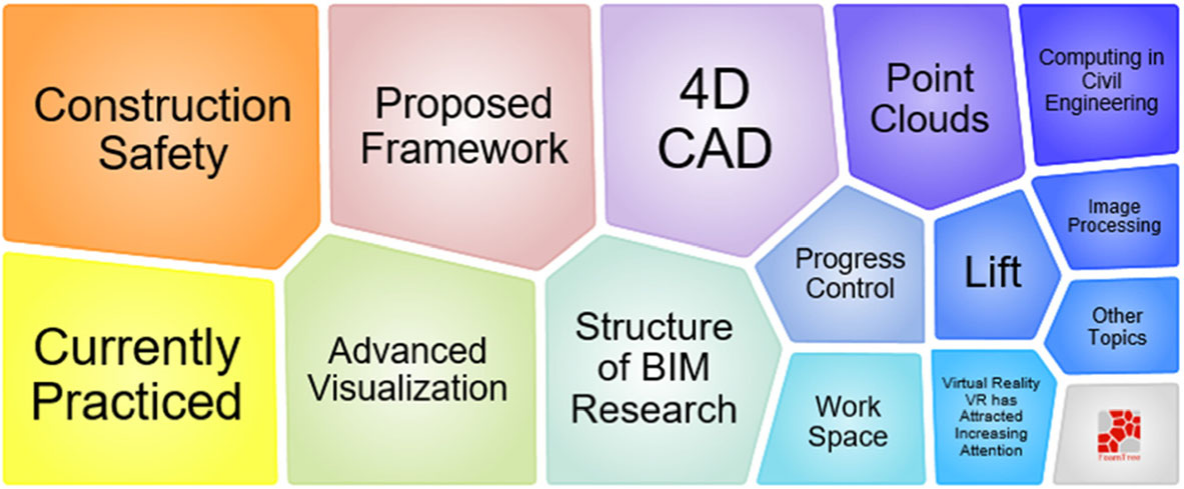
Foam tree view of keywords in topic 2

According to Fig. 10, BIM is mostly used to solve problems related to construction safety and management of the workspace. 4D CAD and point clouds are always related to BIM. The results of BIM related works are mainly proposed framework, technique practice, and the literature review. In Fig. 8, we can observe that topic 2 starts in 2007 and ends in 2016. The most active period is from 2008 to 2014. Although the citation of BIM bursts in recent years, the recent works are not classified in topic 2. Nowadays, similarly to other scientific fields, researchers in visual computing are not satisfied with the use of BIM only, but with quite a lot of new IT techniques.

#### Topic 3: computer vision

This topic is using computer vision to capture and solve construction problems. Several directions appeared, including the automatic object recognition [46–52], automated vision tracking [18, 53–56], vision-based action recognition of specific construction equipment or workers [16, 57–60], vision interpretation [61–63], and using computer vision for specific construction management purposes, such as construction safety and health monitoring [64, 65], progress monitoring [17], and performance monitoring [66]. Also, some sensor techniques were used for monitoring workers’ activities [67] or construction site status [68].

**Fig. 11:**
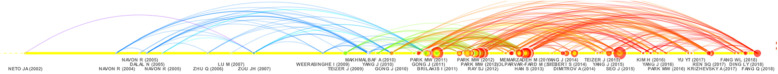
Timeline view of topic 3

Figure 11 shows that topic 3 started in the early 2000s, but highly cited works appeared after 2010. Moreover, in recent years, highly efficient deep learning techniques have brought more possibilities for applying computer vision in construction [60, 69]. Owing to the development of vision-based computational techniques, construction site management has more opportunities to become more automatic and could acquire more real-time site information.

**Fig. 12:**
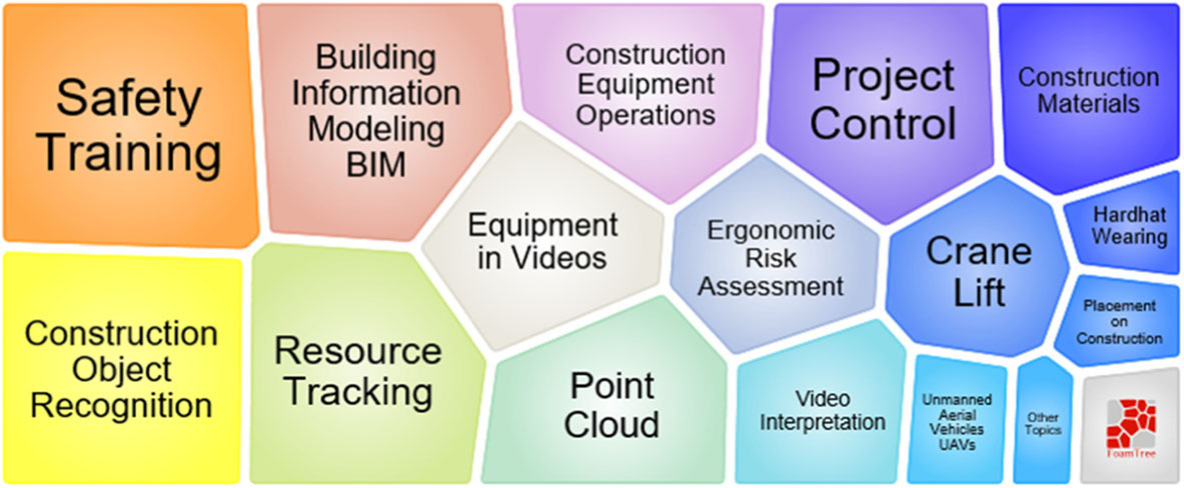
Foam tree view of keywords in topic 3

According to Fig. 12, main construction problems in this topic include safety training, resource tracking, construction equipment operations, risk assessment, project control, and crane lift. Besides, the core techniques related to this cluster are BIM, construction object recognition, equipment video analysis, and point cloud processing.

#### Topic 4: global positioning system

Although topic 4 is tagged as the global positioning system (GPS), the works related to this topic did not only use GPS. It is not clustered well and has two different research directions: location tracking [70, 71] and construction safety [72–75]. Two significantly influent works demonstrate these two directions. The first one uses the ultra-wideband for construction resource location tracking [76]. The second one uses BIM for automatic construction safety checking [77]. Additionally, some works use construction site positioning techniques for construction safety [78, 79].

**Fig. 13:**
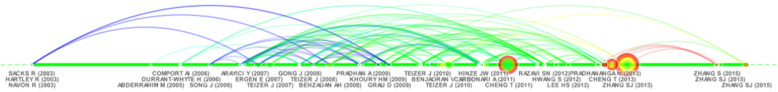
Timeline view of topic 4

Despite presenting two works with significantly high quality, this topic developed more slowly after 2013, as shown in Fig. 13. Two of the three works cited in 2015 are authored by Zhang et al. [77].

**Fig. 14:**
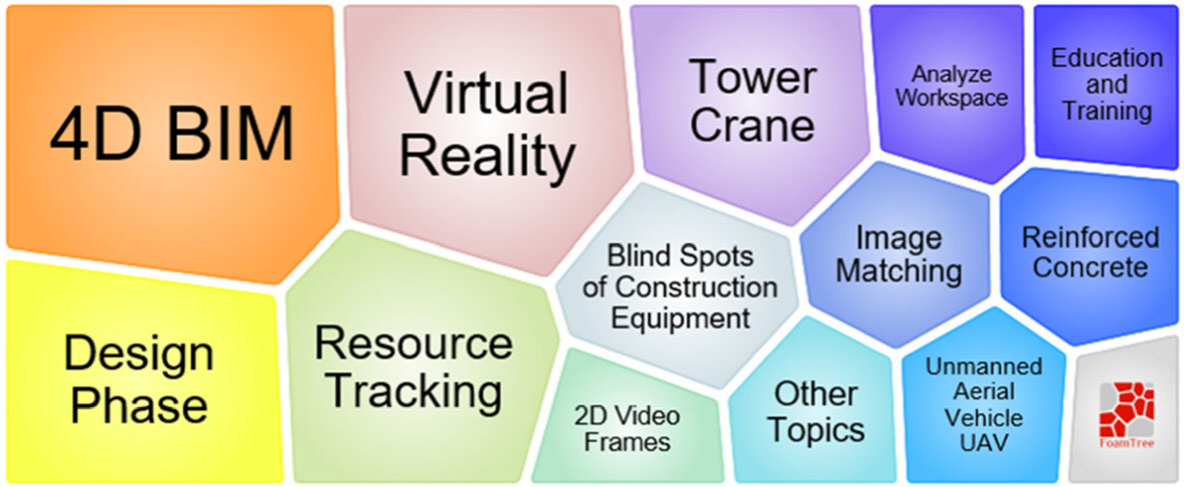
Foam tree view of keywords in topic 4

Figure 14 shows that unlike positioning techniques, 4D BIM, VR, image matching, workspace analysis, and 2D video analysis are commonly used for resource tracking and safety management. Moreover, operation of tower cranes, blind spots of construction equipment, education and training related to safety management are the key topics in this cluster.

#### Topic 5: laser scanning

**Fig. 15:**
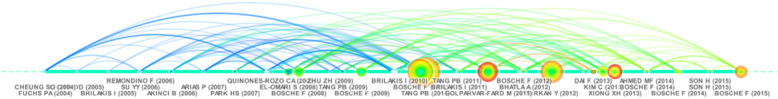
Timeline view of topic 5

The core technique of topic 5 is laser scanning, the most common keywords used in related works. Such works contribute to reconstruction of 3D model from laser-scanned point clouds [80–85], performance analysis of laser scanning [86], and laser scanning for automatic progress tracking [87–89] or quality control [90, 91], or both [92]. Figure 15 shows that this topic started in 2004, and slowly developed for nearly 5 years. In the 2010s, the cluster gained several frequently cited works which have had a continuous influence until today. However, similarly to topic 4, no more works with high impact appeared after 2015.

**Fig. 16:**
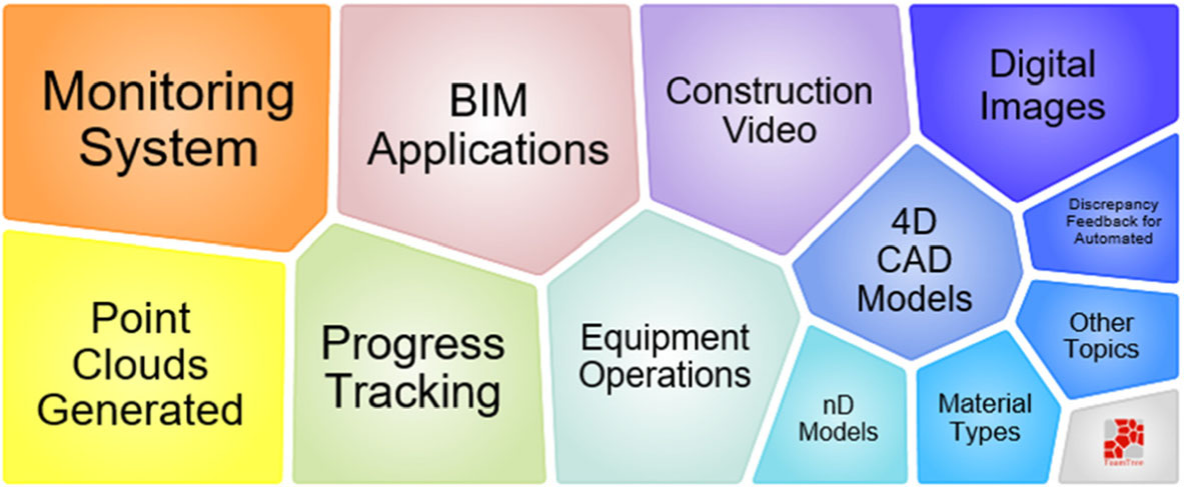
Foam tree view of keywords in topic 5

According to Fig. 16, point clouds generated by laser scanning can be applied to the construction of videos and digital images. As a result, nD CAD or BIM models could be dynamically updated with such kind of technology. Moreover, the most frequent purposes for using laser scanning are progress tracking and monitoring equipment operations.

#### Topic 6: AR

**Fig. 17:**
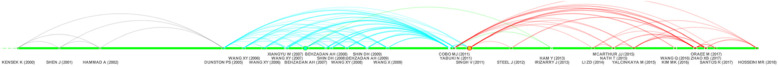
Timeline view of topic 6

AR is well known and has been applied in various engineering fields, including construction. According to Fig. 17, cluster #5 can be divided into three periods. The first is ranging from 2000 to 2005. In this period, it was attempted to apply AR to the AEC industry. In 2005, Dunston and Wang [93] proposed a mixed reality-based visualization interface for the AEC industry, and cluster #5 stepped into a new period. In 2011, Singh et al. [94] proposed a BIM-based system framework for collaboration. Since then, AR was applied with BIM in construction. It is noteworthy that Cobo et al. [95] published a review of science mapping tools in 2011 and was cited by eight works in this topic. Most of the citing works are bibliometric reviews and data research of BIM. Additionally, laser scanning is also covered. Thus, AR is not the only technique in the topic, especially in the third period, since 2011 until now.

**Fig. 18:**
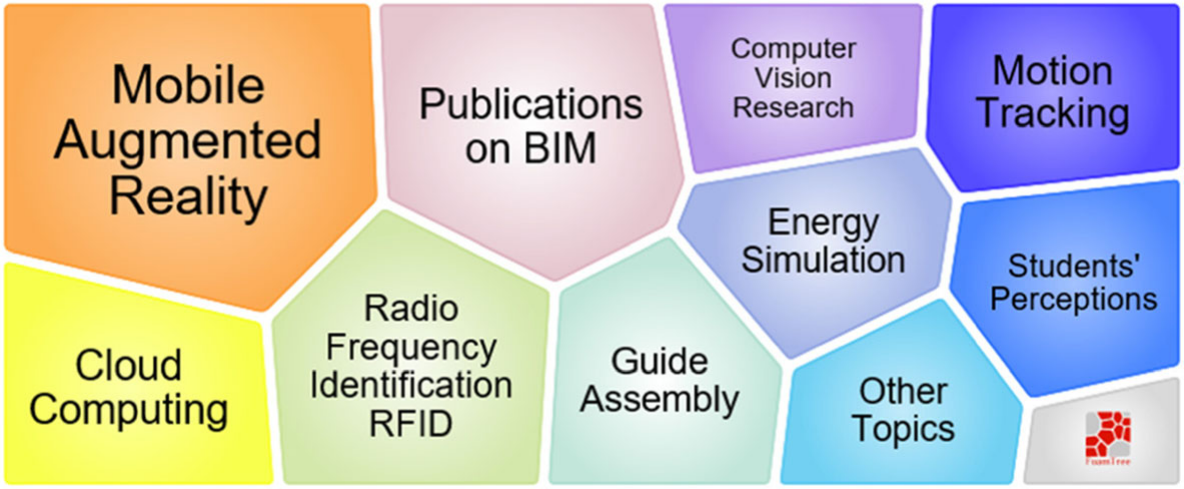
Foam tree view of keywords in cluster #5

Figure 18 indicates why and how to explore the benefits of using AR in construction. Cloud computing, radio frequency identification, BIM, and motion tracking are also introduced with AR for guiding the assembly process and construction education. It is worth mentioning that due to lower equipment costs and easier application approaches, mobile AR makes more sense than traditional AR in construction [96].

#### Topic 7: progress monitoring

**Fig. 19:**
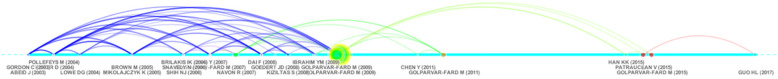
Timeline view of topic 7

Concepts similar to progress monitoring appears in several other topics, such as project control in topic 3 and progress tracking in topic 5. The top-cited work provided by Golparvar-Fard et al. [97] represents the main research direction, which is gathering and using the progress information from visual data. Figure 19 illustrates that the most widely used progress monitoring approach is an integration of 4D simulation model and site photographs [97].

**Fig. 20:**
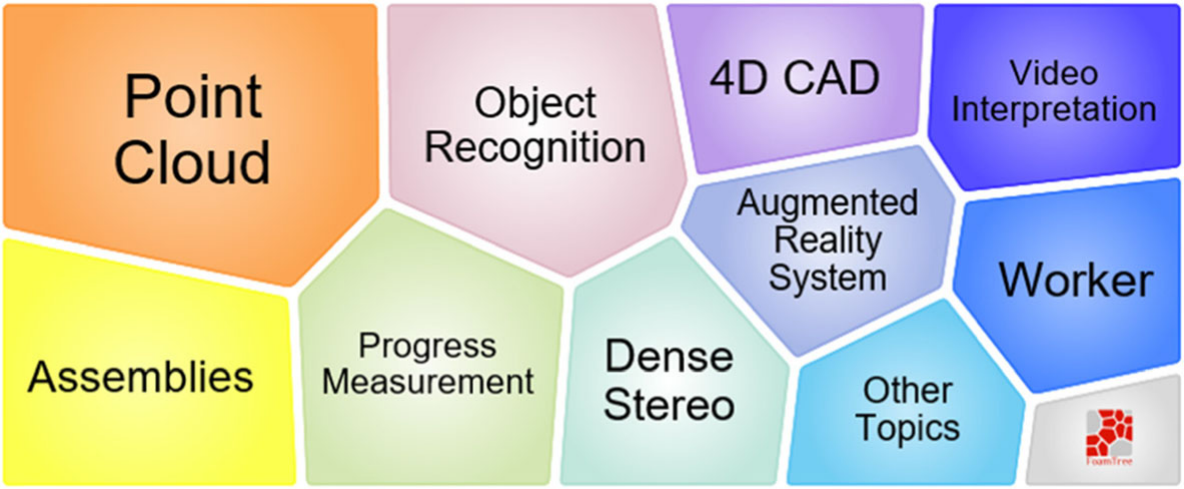
Foam tree view of keywords in topic 7

Figure 20 shows that despite photography being used by the top-cited work, point cloud and video are also used for progress monitoring. Moreover, object recognition, 4D CAD, AR, and dense stereo matching are applied to build the real scene and measure the progress. Most of the works in this topic focus on assembly activities.

#### Topic 8: workspace management

**Fig. 21:**
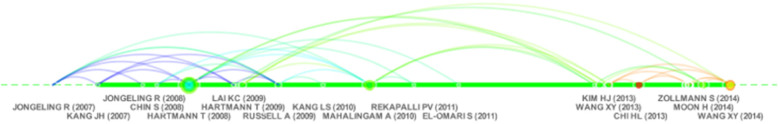
Timeline view of topic 8

**Fig. 22:**
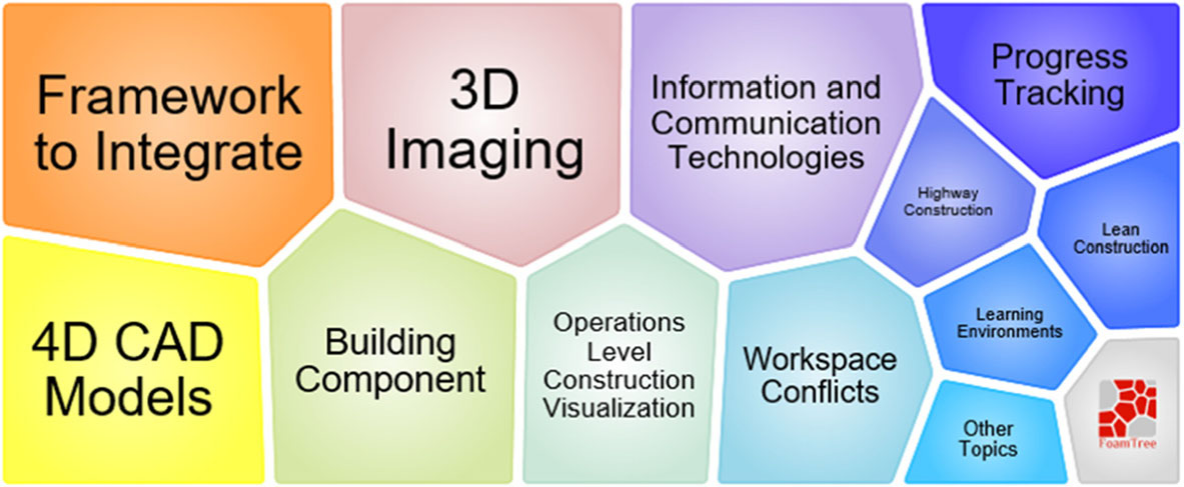
Foam tree view of keywords in topic 8

According to Fig. 21, topic 8 lasts for a short period, from 2007 to 2014. In this topic, 4D CAD [98–100] is the main technique for workspace management. Figure 22 shows that 4D CAD, 3D imaging, construction visualization, progress tracking, and integrating framework are used, demonstrating that workspace management is not the only research purpose. The space analysis approaches are also used in building operations, such as path planning [101].

#### Topic 9: mobile crane

**Fig. 23:**
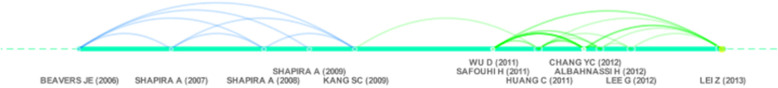
Timeline view of topic 9

**Fig. 24:**
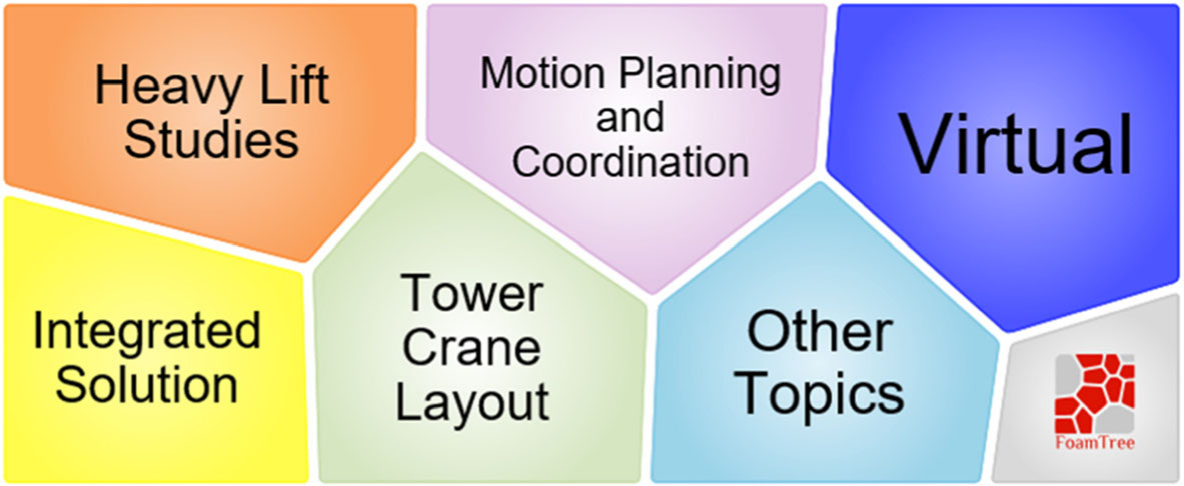
Foam tree view of keywords in topic 9

Topic 9 is from the cluster with the minimum size in the result. According to Fig. 23, from 2006 to 2013, 14 works focused on solving problems such the heavy-lift problem, planning, coordination, and layout, when using a mobile crane on the construction site (Fig. 24). However, compared to other clusters, this cluster attracts less research attention.

## Discussion

The nine topics presented in this study are the main research interests in the field of visual computing in construction. In the topic analysis, the development process, core research direction, and key works associated with each topic are revealed. Most of the suggested labels can represent the core concepts, except for topics 1, 3, and 7. Furthermore, most research topics of visual computing in the search strategy appear in the results, indicating that construction is an inclusive research area for visual computing. However, according to the results, most research directions have reached the bottleneck, including BIM, construction safety, laser scanning, and construction progress control. In recent years, most of the new works in these topics are rarely cited (no cited works after 2016 exactly). Meanwhile, some research topics have been gradually changed or replaced by new research directions, such as 4D CAD, workspace management, and mobile crane. These topics have few works with high attractiveness, and the works which were highly cited in the past have generated fewer citations in recent years.

Some significant findings are listed below:

Most problems related to 3D CAD have been solved and 3D CAD works as a basis in the construction area. No influential work has been published in the last 5 years in the research topic of 3D CAD, and few papers have cited works on this topic recently. Nevertheless, 3D CAD has been the fundamental knowledge of other research topics. 3D CAD provides the edition, storage, handling, and representation methods of 3D geometry information. 3D-related research topics such as BIM, locating, laser scanning, AR, and workspace management frequently cite 3D CAD when they arose. Afterward, BIM gradually replaced 3D CAD as it was developed.

BIM lacks innovative and influential works in the field of visual computing. The developing path of BIM is promising, 15 published works that been cited over ten times (in the scope of this study) have been published. Meanwhile, some works related to BIM are classified in other topics, illustrating that it is highly adaptable. However, there have been hardly new highly cited works recently, while the top-cited works are still being cited. And it is suggested that combination of BIM and other visual computing techniques are interesting for future works.

Computer vision is not widely used and there is an increasing trend in the construction industry. Computer vision-related techniques are used to monitor, recognize, and track construction sites automatically. Additional topic analysis for topic 3 shows the most giant three subtopics in topic 3 are BIM, earthmoving machine, and deep learning. Around 2015, deep learning techniques stepped into a new stage. The improvement of the convolutional neural network [102, 103], extreme learning machine [104], and other deep learning techniques [105] made it possible to recognize objects in image and vision data with high quality and efficiency. Construction site vision analysis techniques, including element and feature recognition, action recognition, vision tracking, vision interpretation, have been studied. Meanwhile, automatic vision processing made it possible to monitor construction safety, progress, and performance. Although some works contribute to the above-mentioned research directions, the improvement of deep learning brings more possibilities. Meanwhile, computer vision is thought to help improving construction monitoring in the future.

Quite a lot of new techniques emerges for object positioning, laser scanning, progress monitoring, safety management. Like BIM, these research topics have been investigated for a couple of years, and highly cited works have emerged. Though different techniques are adopted, the main problems remain the same, for example, object/worker positioning, progress tracking, and safety management are usually mentioned in many literatures. However, these problems still need further investigation and new methods in solving them are welcome.

The above-mentioned analysis results indicate the research trends of visual computing in construction. In this scientific field, computer vision should be the main research topic for the next few years. Despite that other research topics have not generated highly cited works in recent years, it is possible to look for new research directions based on the existing body of knowledge. The researchers working on these topics may also propose new research topics to overcome current scientific limitations.

## Conclusion

This study aimed to review the literature of visual computing in construction to draw the developing history and provide trends for upcoming research works in this area. The conclusions of this article are as follows.

1. 3D design data, point clouds, videos, and photos are primary visual data sources.
2. The topics include techniques of data gathering, handling, analysis, storing, and representation for construction demands such as safety control and schedule management.
3. 3D CAD and BIM are core topics of 3D representation.
4. Most topics have been missing influential works in recent years.
5. Computer vision is suggested as a better topic to publish high-quality papers.

In this paper, the works with significant values are pointed out in each topic to provide a concise way to accumulate knowledge. The study has limitations due to the limited time of data collection and processing, and several potential research directions can be pursued:

1. The search strategy could be more accurate and have a broader coverage.
2. More literature databases and data cleaning techniques could be used to improve the search results.
3. The conclusion could be more convincing if other bibliometric tools and approaches were introduced.
4. It would be worth looking for better cluster results by introducing new bibliometric tools.
5. Topic 8 and topic 9 would be worth further studying, without limitation to this scientific field.

## Data Availability

The datasets used and/or analysed during the current study are available from the corresponding author on reasonable request.

## Abbreviations

2D: Two-dimension
3D: Three-dimension
4D: Four-dimension
AEC: Architecture engineering and construction
AR: Augmented reality
BIM: Building information modeling
CAD: Computer-aided design
GPS: Global positioning system
